# *Wolbachia* infection confers post-translational modification of glutamic acid decarboxylase and other proteins in *D. melanogaster*

**DOI:** 10.1128/spectrum.02465-24

**Published:** 2025-04-28

**Authors:** Sarah M. Boothman, Sarah Preston, Jonathan Minden

**Affiliations:** 1 Department of Biological Sciences, Carnegie Mellon University 171620, Pittsburgh, Pennsylvania, USA; Brigham Young University, Provo, Utah, USA

**Keywords:** *Wolbachia*, *Drosophila*, glutamic acid decarboxylase

## Abstract

*Wolbachia pipientis* is a ubiquitous intracellular bacterium that is known for its manipulation of reproduction in arthropod hosts. *Wolbachia* has also been shown to colonize virtually all somatic tissues, including the brain, but little is known about the interaction between host and bacterium in these locations. To this end, we studied the effects of *Wolbachia* infection on the brain of *Drosophila melanogaster*. Using comparative proteomics, we uncovered the post-translational modification of many proteins within the *Drosophila* head and body upon infection, with glutamic acid decarboxylase being modified within the head only. Given this enzyme’s role in neurotransmitter synthesis, we next tested how *Wolbachia* infection impacts host behaviors and gamma aminobutyric acid (GABA) production within *Drosophila*. We discovered an improved response to yeast odors in *Wolbachia*-infected, mated females compared with their uninfected counterparts. Gross measurements of GABA in whole brains showed no detectable change in GABA abundance upon infection. Treatments with a GABA antagonist indicated that the behavioral change was not GABA-dependent, leaving the mechanism behind *Wolbachia*-mediated changes in behavior obscure. Given the multiple protein changes in the *Drosophila* head upon infection, we propose a model in which *Wolbachia* drives the modification of glutamic acid decarboxylase and several metabolic proteins to increase survival in the specialized niche of the brain. These results give rise to new questions about the *Wolbachia–Drosophila* relationship, and future work will focus on the mechanism through which *Wolbachia* confers these protein changes.

**IMPORTANCE:**

In order to fully understand the biology of an organism, we must understand its interactions with its resident microbes. *Wolbachia* is commonly used to study such interactions, but the molecular interactions this bacterium has with its hosts are not well understood, especially within somatic tissues. Here, we address this knowledge gap by characterizing the changes in host proteins within *Drosophila melanogaster* upon *Wolbachia* infection. Our results provide the first description of post-translational modifications induced by *Wolbachia* infection within a host, unveiling a new level of regulation in the *Wolbachia*–host relationship. The modification of glutamic acid decarboxylase within the *Drosophila* head was not shown to be connected to changes in GABA production or host behavior, indicating another role for this enzyme during *Wolbachia* infection within the brain. Altogether, these results provide more information about *Wolbachia*’s infection of somatic tissue and spark new inquiries into the host–bacterium relationship.

## INTRODUCTION

*Wolbachia pipientis* (commonly shortened to *Wolbachia*) is an intracellular alpha-proteobacterium that is spread vertically throughout insect populations. It is considered one of the most successful endosymbionts in the world, as it is present in approximately 40% of arthropod species (1). The relationship it has with its hosts ranges from mutualistic to parasitic, and the molecular basis of this wide variety of interactions is of particular interest in the symbiosis field (2). For decades, the obligate intracellular nature of *Wolbachia* made it a difficult organism to study, but recent advances in molecular and biochemical techniques have made it possible to probe the mechanisms used by this bacterium to manipulate its hosts (3–5).

Much of what is known about *Wolbachia* biology comes from work in *Drosophila*. Many studies focus on the bacterium’s manipulation of the germline in order to ensure its successful transmission to new generations (3, 6). Notably, *Wolbachia* has also been shown to colonize multiple somatic tissues within *Drosophila*, such as the gut, fat bodies, and the central nervous system (5, 7). These sites are seen as a “dead end” for the microbe because it will not be transferred to new hosts from these tissues. Even so, the presence of the bacterium in these locations poses the opportunity to provide benefits to and/or manipulate its host to sustain colonization. Investigation into the basic host*–Wolbachia* interactions within these tissues will elucidate potential advantages for microbial localization to these areas while also broadening our understanding of the host*–Wolbachia* relationship.

Localization to the adult brain of *Drosophila* has been shown to be a conserved feature of *Wolbachia* infection (7, 8). The brain is a surprising niche for these bacteria, given the specialization and high metabolic needs of the host cells that reside there (9). One prevailing theory for the localization of *Wolbachia* within the brain follows the “Behavioral Manipulation Hypothesis,” in which a microbe alters host behavior to benefit its own transmission (6). Accordingly, *Wolbachia* infection has been correlated with multiple changes in fundamental *Drosophila* behaviors, including differential mating choices, reduced aggression, increased sleep, improved olfactory response, and enhanced learning and memory capacity (10–14). However, it is unclear if these effects are a direct or indirect result of *Wolbachia* infection, let alone colonization of the brain. By studying the molecular interactions of the bacterium within neural tissue, we can gain insight into how these changes in behavior are produced by infection, which will further inform how *Wolbachia* interacts with somatic tissues on a molecular level.

Recent advances in sequencing and bioinformatics have enabled molecular study of the *Wolbachia*-host interaction through various -omic approaches(15–23). Only a few studies have utilized proteomics to study *Wolbachia*-induced changes in the host (18–20). Proteomics adds another level to the knowledge of *Wolbachia*-host interactions and provides a more comprehensive view of the molecular effects of infection. These investigations yield data about differences in protein levels upon infection, but also changes in protein modification and interactions, all of which play a role at the bacteria–host interface (24). All previous studies have focused on protein abundance changes within reproductive tissues and in cell lines(18–20). We aim to expand upon these inquiries by investigating protein changes within somatic tissues and by investigating protein modifications upon *Wolbachia* infection in these tissues.

Here, we performed a proteomic screen of *D. melanogaster* heads infected with the *Wolbachia* strain *w*Mel to identify how proteins change upon infection. In addition to changes in various metabolic proteins, we observed a post-translational modification of glutamic acid decarboxylase (GAD) in infected flies. We then explored the consequences of this modification in the *Drosophila* brain by assaying changes in olfactory behavior and gamma aminobutyric acid (GABA) levels in infected and uninfected flies. Given previous findings that GABA can be used as a metabolic intermediate by microbes (25), we propose a model in which *Wolbachia* influences the production of GABA within neurons of the *Drosophila* brain in order to compensate for the lack of metabolic intermediates available for scavenging from the host. These findings add to the understanding of how *Wolbachia* interacts with its *Drosophila* host on a molecular level and open new pathways of inquiry into how microbes adapt to specialized niches, such as the brain.

## RESULTS

### Two-dimensional difference gel electrophoresis (2D-DIGE) reveals protein changes in *D. melanogaster* heads and bodies upon *Wolbachia* infection

**Fig 1:**
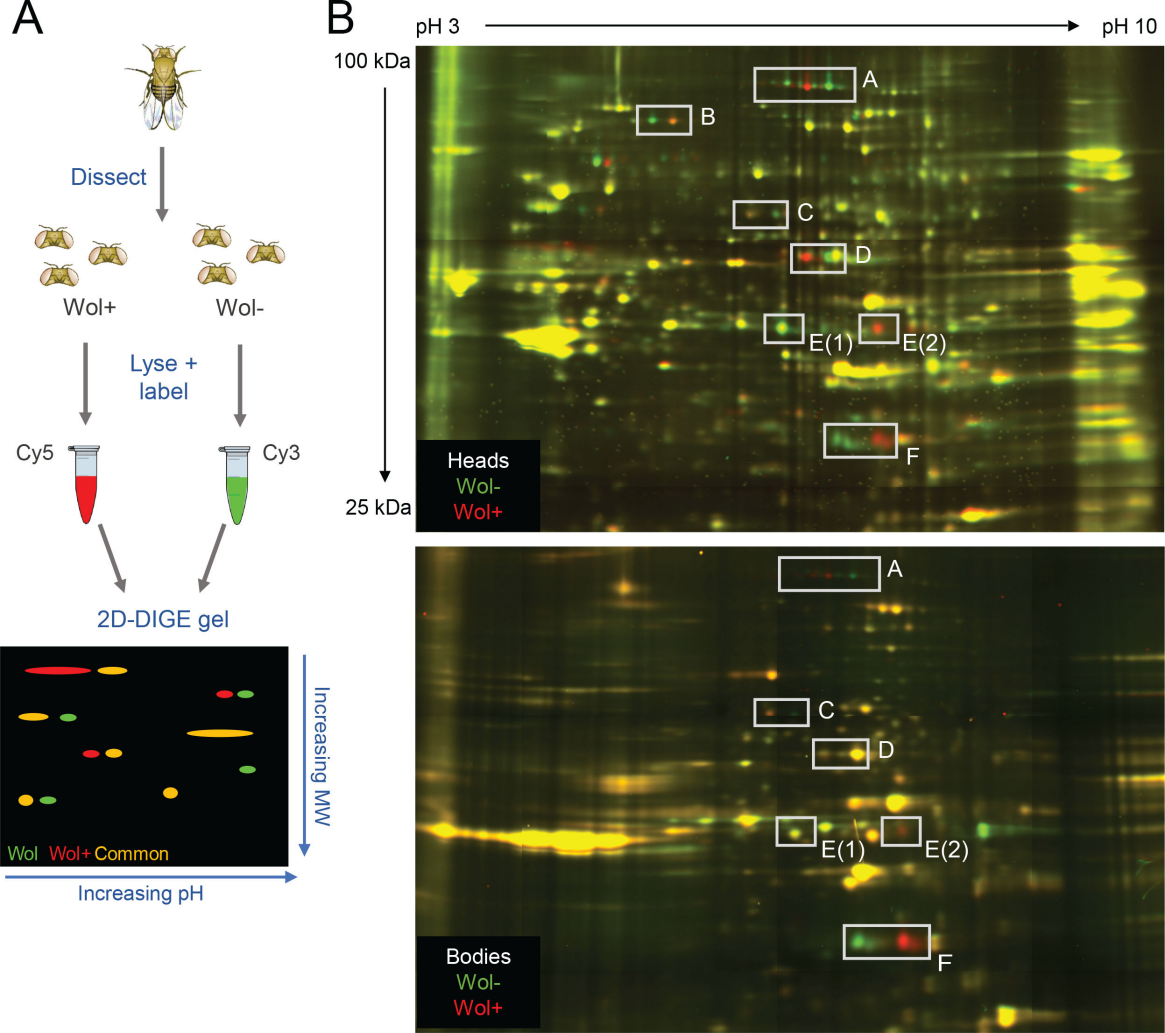
Two-dimensional difference gel electrophoresis (2D-DIGE) reveals protein changes in *D. melanogaster* heads and bodies upon *Wolbachia* infection. (**A**) Schematic of 2D-DIGE experiment to compare protein extracted from uninfected and infected *D. melanogaster*. (**B**) Resultant gel images of 2D-DIGE analysis of *Wolbachia*-infected and uninfected fly heads (top) and decapitated bodies (bottom). Wol + protein is labeled with Cy5 (red), and Wol− protein is labeled with Cy3 (green). Proteins that showed consistent changes across multiple gels are indicated, and labels correspond to protein identifications found in Table 1. Vertical and horizontal lines in the gel images are artifacts of the image acquisition methods used for these gels (see Materials and Methods).

To detect changes in the proteome of *D. melanogaster* upon infection with the *Wolbachia* strain *w*Mel, 2D-DIGE analysis (26) was performed (Fig. 1A). When protein from infected and uninfected fly heads was compared, multiple differences were observed (Fig. 1B, top). We identified six protein spots that consistently changed in *D. melanogaster* heads across biological and technical replicates (labeled A–E in Fig. 1B). Interestingly, all selected proteins displayed horizontal shifts on the 2D gel, representing a change in isoelectric point without a change in mass, which is typical of a post-translational modification (PTM). Spots A, C, and D appeared to shift to the left upon *Wolbachia* infection, indicating the proteins became more acidic. Conversely, spots B, E, and F shifted to the right upon infection, suggesting they became more basic. The leftmost and rightmost forms of the proteins were excised from the 2D gels and identified via liquid chromatography–tandem mass spectrometry (LC-MS/MS) (Table 1). The left and right forms of all candidate spots were identified as the same protein, indicating that these proteins are indeed undergoing modification during *Wolbachia* infection.

**TABLE 1:** Identification of *D. melanogaster* proteins differentially modified during *Wolbachia* infection via LC-MS

Protein ID	Accession number	Gel spot*^a^*		Number of peptides	Sequence coverage
Glycogen phosphorylase	Q9XTL9	A	Left	7	11.4%
			Right	5	7.3%
Iron regulatory protein 1B	Q9VGZ3	B	Left	14	18.4%
			Right	22	27.5%
Inositol-3-phosphate synthase	O97477	C	Left	19	39.5%
			Right	27	54%
Glutamic acid decarboxylase	P20228	D	Left	28	51.6%
			Right	18	32.5%
Elongation factor 1-gamma	Q9NJH0	E	Left	7	22.5%
			Right	8	24.1%
Glycerol-3-phosphate dehydrogenase (cytosolic)	P13706	F	Left	23	72.7%
			Right	25	78%

^*a*^ Protein spots that consistently changed across gels in 2D-DIGE analysis (Fig. 1). “Left” and “right” correspond to the leftmost and rightmost form of each spot on the gel.

To quantify the protein changes observed via 2D-DIGE, gel spots were quantified by measuring total fluorescence intensity using S-Extractor software (27). The intensity of Cy3 and Cy5 fluorescence in each spot was normalized against unchanging “guide star” proteins in the same gel, allowing us to calculate the ratios of Wol + to Wol− protein in each protein spot. These ratios were log_2_ transformed to yield an output where protein forms more abundant in Wol+ conditions had a positive value and protein forms more abundant in Wol− conditions had a negative value (Table 2). The results of these calculations confirm our qualitative observations in that all proteins had at least one form more abundant in Wol + heads and at least one form more abundant in Wol− heads. The most extreme difference was observed in glycerol-3-phosphate dehydrogenase (GPDH, spot F), in which the rightmost forms of the protein had a normalized ratio greater than four and the leftmost forms of the protein had a normalized ratio of approximately −2. All selected spots showed a significant difference in normalized ratios of Wol + and Wol− protein between their rightmost and leftmost spots. Because *Drosophila* have been shown to exhibit sexual dimorphisms in the expression and post-transcriptional regulation of genes (28, 29), we also quantified these changes in 2D-gels of male, mated female, and virgin female head lysates, but found that proteins in all three groups were affected by *Wolbachia* infection in the same manner (Table S1).

**TABLE 2:** Quantification of protein from *Wolbachia*-infected and uninfected *D. melanogaster* samples in spots identified by 2D-DIGE analysis

Protein ID	Gel spot^*a*^		Log_2_(Wol+/Wol−)^*b*^			
			Head average ± SE	*P*-value^*c*^	Body average ± SE^*d*^	*P*-value^*c*^
Glycogen phosphorylase	A	1	1.79 ± 0.54	2.35 × 10^−4^	1.31 ± 0.60	3.81 × 10^−4^
		2	1.37 ± 0.30		1.76 ± 0.57	
		3	−1.26 ± 0.42		−1.92 ± 0.81	
		4	−2.73 ± 0.39		−2.34 ± 0.71	
Iron regulatory protein 1B	B	1	−2.09 ± 0.50	3.29 × 10^−4^	*	-
		2	1.04 ± 0.16		*	
Inositol-3-phosphate synthase	C	1	0.66 ± 0.13	6.12 × 10^−5^	0.45 ± 0.13	0.010
		2	−1.57 ± 0.16		−2.53 ± 0.95	
Glutamic acid decarboxylase	D	1	1.76 ± 0.10	3.67 × 10^−5^	0.11 ± 0.01	0.153
		2	−0.57 ± 0.06		0.006 ± 0.07	
Elongation factor 1-gamma	E	1	−1.37 ± 0.20	1.09 × 10^−7^	−0.71 ± 0.28	0.012
		2	2.05 ± 0.15		1.33 ± 0.34	
Glycerol-3-phosphate dehydrogenase	F	1	−2.04 ± 0.35	6.83 × 10^−6^	−0.95 ± 0.17	1.22 × 10^−7^
		2	−1.96 ± 0.29		*	
		3	4.17 ± 0.63		2.32 ± 0.12	
		4	4.55 ± 1.43		*	

^*a*^ Protein spots that consistently changed across gels in 2D-DIGE analysis. A spot number of 1 corresponds to the leftmost form of the protein on the gel, and position moves to the right as spot number increases.

^*b*^ Ratio of protein from Wol+ samples and Wol− samples in the spot, calculated from quantification of Cy3 and Cy5 intensities on 2D-DIGE gels using S-Extractor software. N ≥ 4 gel images for each spot.

^*c*^ *P*-value calculated via Student’s t-test comparing log2 ratios of the rightmost and leftmost spots of a given protein. -, comparison not performed due to poor resolution on gels.

^*d*^ *, protein spot unable to be quantified due to poor resolution on gels.

To test whether these *Wolbachia*-induced protein changes were specific to the head, 2D-DIGE analysis was performed on the decapitated bodies of infected and uninfected *D. melanogaster* (Fig. 1B, bottom). Almost all the identified proteins from the 2D gels of head lysates were observed in the gels of body lysates. The one exception was iron regulatory protein (IRP1B, spot B), but this result could be due to poor resolution of proteins from body lysates in the region of the 2D gels where IRP1B migrates. Of the proteins observed on the gels of body lysates, all displayed the same horizontal shift as on the gels of head lysates, and most displayed the same Wol + and Wol− protein patterns (Fig. 1B). Almost all spots also showed normalized Wol+/Wol− ratios similar to those in the head, indicating *Wolbachia* infection is inducing the same changes in these proteins in both the heads and the bodies of *D. melanogaster* (Table 2).

**Fig 2:**
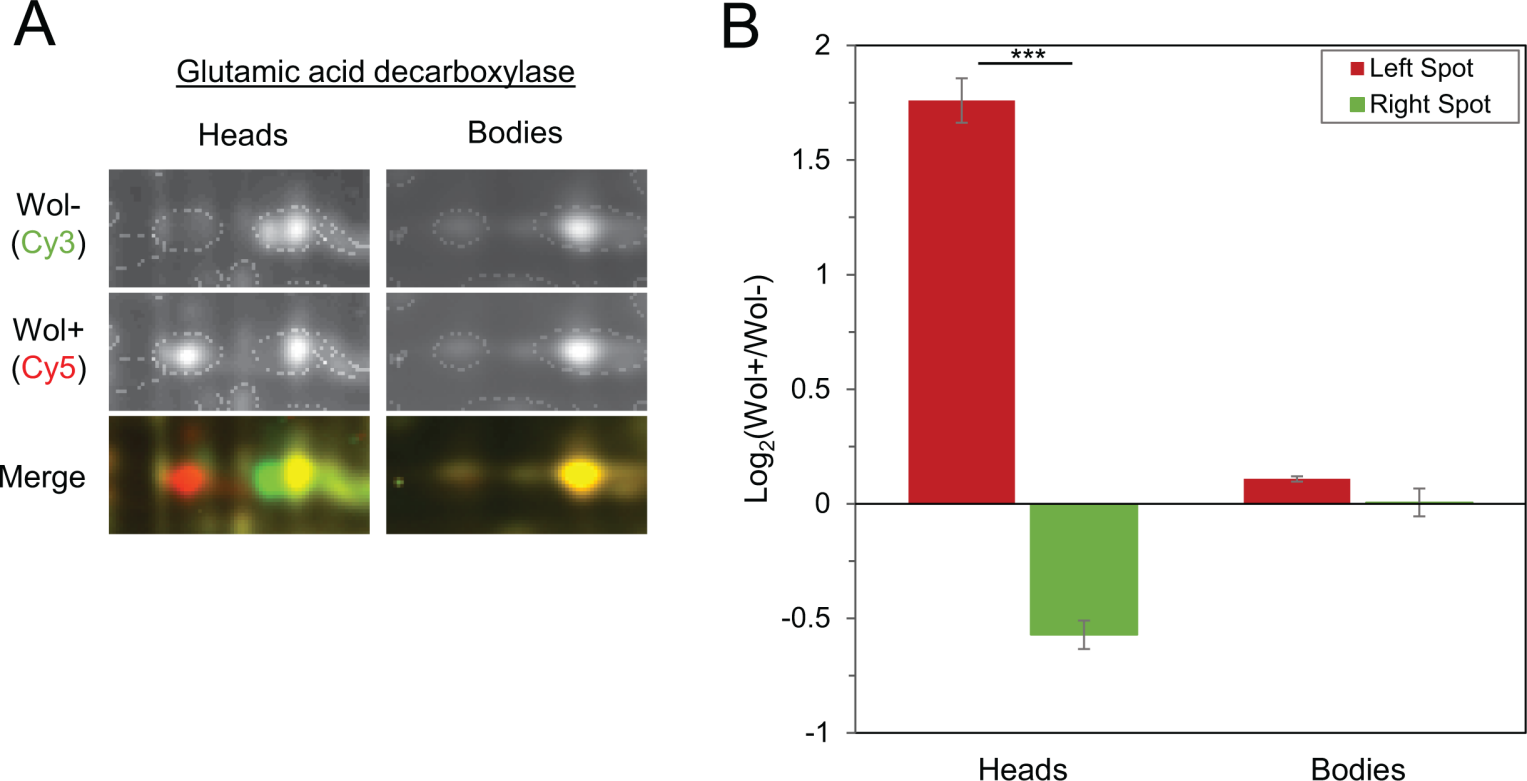
Glutamic acid decarboxylase is modified in the heads, but not bodies, of *D. melanogaster* upon infection with *w*Mel. (**A**) Cropped image of the 2D-DIGE protein spots for GAD. The horizontal shift in the protein indicates a change in isoelectric point, which is indicative of a post-translational modification. (**B**) Comparison of normalized Wol + to Wol− protein ratios in each GAD spot from 2D-DIGE analysis of fly heads and bodies. *N* = 4 gel images; ***P < 0.0005.

Interestingly, glutamic acid decarboxylase (GAD, spot D) was the only exception to these observations. The GAD protein spot displayed a shift to the left upon infection in heads but did not appear to change in the body of *D. melanogaster* (Fig. 2A). Upon quantification of these spots, a significant difference in Wol+/Wol− ratio between the left and right GAD spots in the head was found (Fig. 2B, *P* = 3.67×10^−5^). In contrast, the Wol+/Wol− ratios for both GAD forms in the fly body were very low, with the right spot showing virtually equal amounts of Wol + and Wol− protein. Furthermore, there was no significant difference between the ratios of the left and right GAD spots in the body (*P* = 0.153). Taken together, these data suggest that while *Wolbachia* infection leads to the modification of multiple proteins throughout the fly, GAD is modified specifically within the head.

### Overall GABA levels do not change in *D. melanogaster* brains upon *Wolbachia* infection

**Fig 3:**
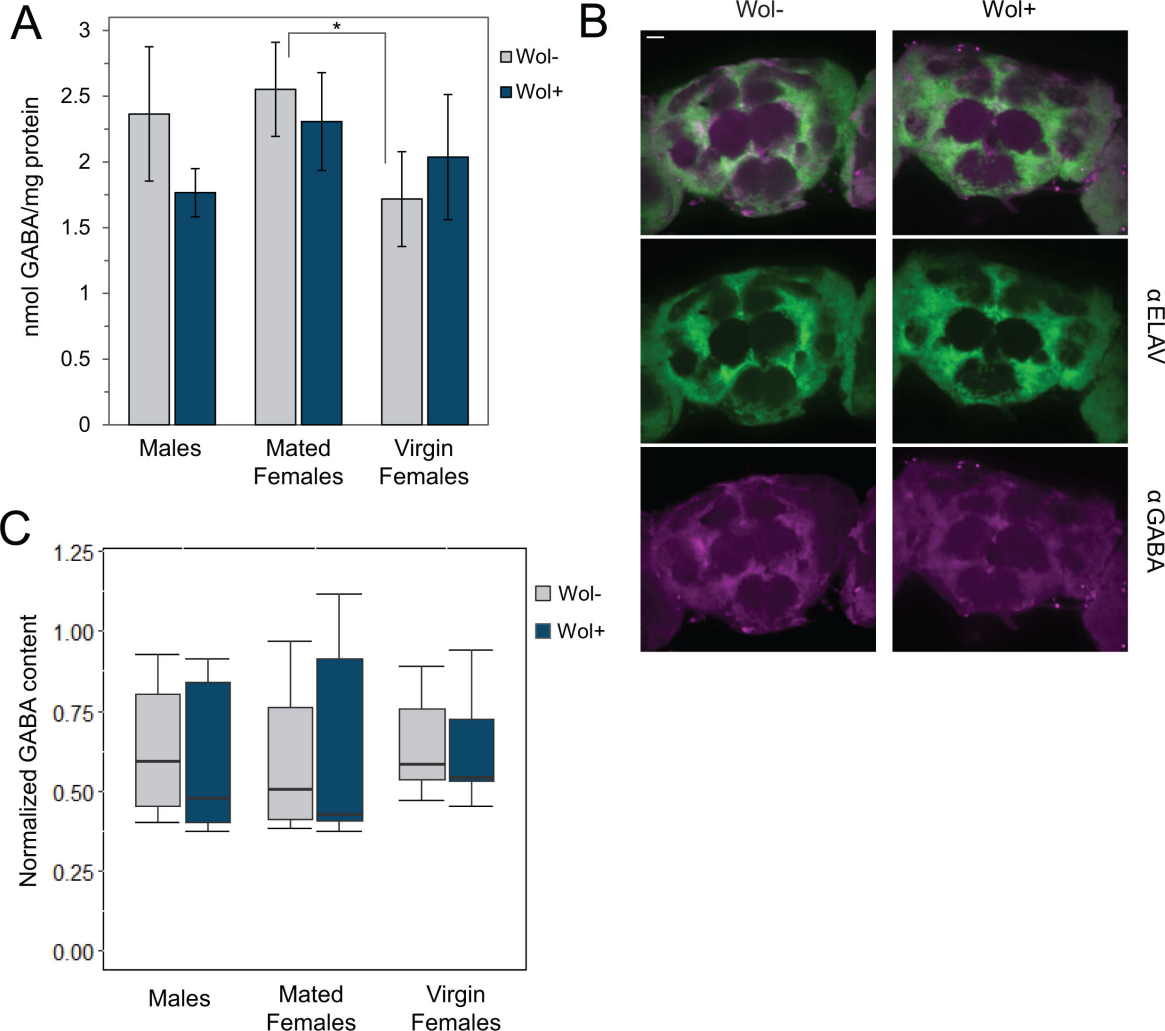
GABA levels do not change overall upon *Wolbachia* infection in *D. melanogaster*. (**A**) Comparison of GABA content in *Wolbachia*-infected and uninfected head lysates (normalized to the protein levels). *N* = 5 replicates of 100 heads each; error bars represent SEM. (**B**) Representative images of GABA immunostaining of w^1118^ brains with and without *Wolbachia*. Brains were also stained with an antibody against ELAV to visualize neurons. Images are Z-projections of approximately 50 µM stacks. Scale bar = 25 µM. (**C**) Quantification of GABA staining in immunostained brains, separated by sex and mating status. GABA intensity was normalized against ELAV staining for each brain. *N* ≥ 8 for each group, *P < 0.05.

To determine whether modification of GAD during infection changes the levels of GABA within the brain, we first employed a fluorescence-based enzymatic assay to measure GABA concentrations in *Wolbachia*-infected and uninfected heads. We were able to detect GABA in all head lysates, with all samples having between 1.5 and 2.5 nmol of GABA per mg protein (Fig. 3A). There was no significant difference found in GABA levels of *Wolbachia*-infected and uninfected heads. We did observe an increase in GABA levels within the heads of uninfected mated females compared with uninfected virgin females, but this difference was not present in the infected females (*P* = 0.014 and 0.264 for Wol− and Wol+, respectively). Ultimately, these data were highly variable, leaving conclusions about GABA production in the *Wolbachia*-infected brain incomplete.

To further quantify how GABA production changes during infection, we employed immunostaining of infected and uninfected brains to visually assess GABA levels (Fig. 3B). We observed GABA staining throughout the brain, with more concentrated regions close to the antennal lobe. There was no obvious difference in GABA content of infected and uninfected brains. To quantify these observations, we measured the GABA intensities within each brain and normalized against the intensity of the neuronal marker ELAV (Fig. 3C). We found that there was no significant difference in overall GABA content in the brains of infected and uninfected *D. melanogaster* (*P* = 0.235, 0.423, and 0.206 for males, mated females, and virgin females, respectively, by Student’s *t*-test). Additionally, levels of GABA were not shown to be affected by mating status or sex of the flies (*P* > 0.05 for all comparisons).

### Olfactory behavior changes in males and mated females upon *Wolbachia* infection

To uncover whether the change in GAD upon *Wolbachia* infection is related to changes in *Drosophila* behavior upon infection, we examined GABA-related behaviors in infected and uninfected flies. The olfactory response in *Drosophila* is known to be governed by GABAergic circuits (30, 31) and has also been shown to be perturbed upon *Wolbachia* infection (13, 32, 33). Thus, we assessed the olfactory response of *Wolbachia*-infected and uninfected flies using odor traps baited with yeast paste. We also measured the general activity of flies within these assays using unbaited control traps to ensure the effects seen in these experiments were a result of olfactory, not locomotor, changes. Males, mated females, and virgin females were tested separately. Males and mated females with *Wolbachia* showed higher capture proportions than their uninfected counterparts in yeast-baited traps over time (*P* = 1.2 × 10^−4^ and 5.7 × 10^−10^ by Cox proportional hazard model for males and mated females, respectively), where infection did not significantly affect capture of virgin females (*P* = 0.948) (Fig. S1, Table S2). However, capture rates in unbaited traps were significantly increased upon infection for all groups (*P* = 6 × 10^−6^, 0.001, 0.006 for males, mated females, and virgins, respectively) (Fig. S1, Table S2). Thus, it is unclear if the effect seen in baited traps is motivated by olfactory changes or differences in locomotion.

**Fig 4:**
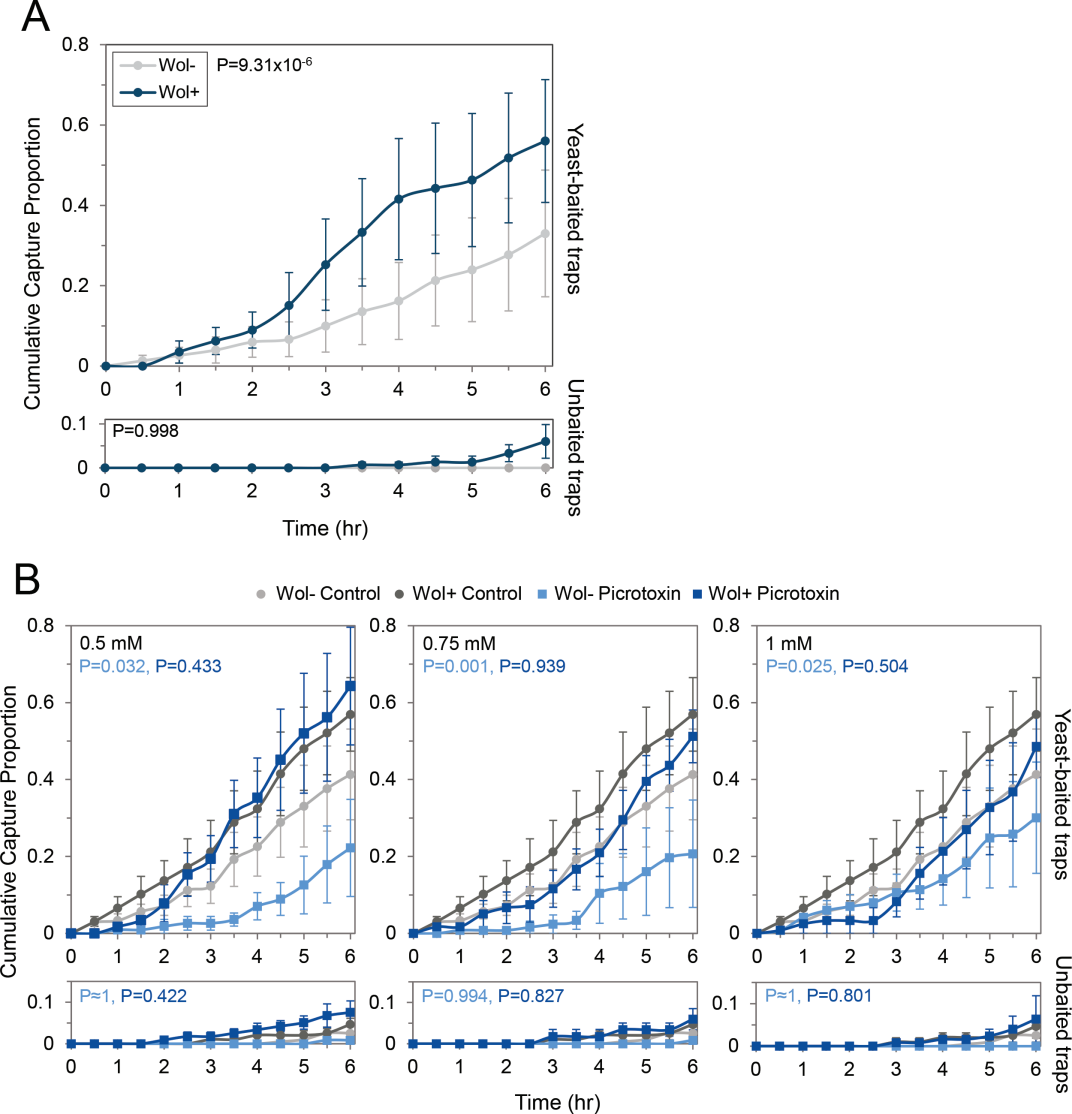
Olfactory response to yeast odors changes in mated *D. melanogaster* females upon *Wolbachia* infection but not in a GABA-dependent manner. (**A**) Cumulative proportion of mated female flies (both infected and uninfected with wMel) captured in an odor-baited trap assay. Flies were placed in an arena containing a yeast-baited trap, and the number of flies caught in the trap was scored in 30 min intervals (top). Capture activity of flies in unbaited control traps was also assessed (bottom). Significant differences between curves were determined via a Cox proportional hazard model. (**B**) Cumulative proportion of mated females captured in the odor-baited trap assay following treatment with the GABA_A_ receptor antagonist picrotoxin. *P*-values displayed were calculated using a binomial GLM for interaction between infection status and treatment type (see Table 3). *N* = 5 trials with 25 flies each; error bars represent SEM.

When we focused our analysis on the first 6 h of the assay, males and mated females still showed a significant increase in capture rate upon infection in yeast-baited traps (*P* = 0.013 and 9.3 × 10^−6^ for males and mated females, respectively), but Wol− and Wol + flies in both groups no longer showed a significant difference in capture in unbaited traps (*P* = 0.998 for both males and mated females; Table S1). Virgin females continued to demonstrate increased capture in unbaited traps (*P* = 0.003) and no significant difference in yeast-baited capture upon infection (*P* = 0.062; Table S2). Therefore, we can confidently assess the olfactory response of males and mated females but not virgin females within our assay. Due to the higher overall response of mated females in the olfactory assay (33% and 56% of mated females captured compared to 2% and 11% of males captured at hour six for Wol− and Wol + flies, respectively), we focused the rest of our analysis on this population. Mated females with *Wolbachia* show an increased capture rate compared to Wol-mated females in yeast-baited traps, but not unbaited traps, within the first 6 h of the assay, indicating that *Wolbachia* infection is affecting the olfactory response to yeast odors within these flies (Fig. 4A).

### *Wolbachia*-dependent olfactory changes are refractory to GABA_A_ receptor inhibition

To assess whether the improvement of olfactory response upon *Wolbachia* infection was dependent on GABA signaling, we treated mated females with the GABA_A_ receptor antagonist picrotoxin. Control-treated groups showed a similar capture rate in yeast-baited traps to that of preliminary untreated assays, with approximately 40% and 60% of Wol− and Wol + flies being captured within the first six h of the assay, respectively (Fig. 4B, top panels). Capture proportions in unbaited traps were also similar to untreated assays, with approximately 2% and 5% of control-treated Wol− and Wol + flies, respectively, captured at hour 6 (Fig. 4B, bottom panels). Additionally, we observed the same trend in all treatment groups as in previous assays, with Wol + flies generally showing higher capture proportions than their Wol− counterparts in yeast-baited traps (*P* = 0.001 for all Wol− vs Wol + by GLM, Table 3).

**TABLE 3:** Results of the generalized linear model assessing the effects of *Wolbachia* infection and treatment with picrotoxin on the capture of *D. melanogaster* in odor-baited trap assays

Trap type	Predictor	Coefficient	SE (Coef)	Z-value	*P*-value
Yeast baited	Intercept	24.4599	2.2849	10.705	2 E-16
	Time	−4.3897	0.3861	−11.369	2 E-16
	Infection	0.6832	0.214	3.192	0.00141
	0.5 mM treatment	−0.267	0.2869	−0.931	0.35209
	0.75 mM treatment	−0.6817	0.298	−2.288	0.02214
	1 mM treatment	−0.3237	0.2887	−1.121	0.26208
	Uninfected: 0.5 mM treatment	−0.8618	0.40332	−2.137	0.03262
	Uninfected: 0.75 mM treatment	−1.5632	0.4794	−3.261	0.00111
	Uninfected: 1 mM treatment	−1.00794	0.44934	−2.243	0.02489
	Infected: 0.5 mM treatment	0.32176	0.41085	0.783	0.43354
	Infected: 0.75 mM treatment	0.03059	0.39885	0.077	0.93887
	Infected: 1 mM treatment	0.26044	0.39012	0.668	0.5044
Unbaited	Intercept	279.3734	18846.21	0.015	0.988
	Time	−47.6085	3141.035	−0.015	0.988
	Infection	17.8171	2149.438	0.008	0.993
	0.5 mM treatment	−0.9129	1.1237	−0.812	0.417
	0.75 mM treatment	0.454	0.7159	0.634	0.526
	1 mM treatment	0.1898	0.772	0.246	0.806
	Uninfected: 0.5 mM treatment	−0.2429	3141.619	~0	~1
	Uninfected: 0.75 mM treatment	16.8335	2149.438	0.008	0.994
	Uninfected: 1 mM treatment	−0.9704	5073.171	~0	~1
	Infected: 0.5 mM treatment	−0.9036	1.1247	−0.803	0.422
	Infected: 0.75 mM treatment	0.1676	0.7733	0.217	0.828
	Infected: 1 mM treatment	0.195	0.7735	0.252	0.801

Generally, we observed a decrease in capture proportion in yeast-baited traps for picrotoxin-treated flies compared to control-treated flies (Fig. 4B, top panels). Capture rates of Wol− flies appeared to have a more drastic decrease than that of their infected counterparts. For example, treatment with 0.75 mM picrotoxin caused the final capture proportion of Wol− flies to decrease from 41% in the control condition to 20% in treated flies, while Wol + capture proportions only slightly dropped from 57% in controls to 51% in treated flies. Additionally, treatment with the antagonist did not appear to affect the capture of flies in unbaited traps (Fig. 4B, bottom panels). For example, the final capture proportion changed from 2% in controls to 1% after 0.75 mM treatment in Wol− flies, and the capture proportion of Wol + flies was approximately 5% for both control and treated flies.

We employed a binomial generalized linear model (GLM) to identify how picrotoxin treatment and *Wolbachia* infection affected the capture of flies in these experiments (Table 3). When treatment with picrotoxin is assessed generally across both infection types in yeast-baited traps, all concentrations were predicted to negatively impact capture compared with the control, but only the 0.75 mM concentration showed a significant effect (*P* = 0.352, 0.022, and 0.262 for 0.5, 0.75, and 1 mM, respectively; Table 3). To determine how infection status affects picrotoxin’s effect on capture rates, we utilized a GLM with infection and treatment as interacting factors (Table 3). All three concentrations of picrotoxin were modeled to have a negative impact on the capture proportion of uninfected flies compared with control-treated flies (*P* = 0.032, 0.001, and 0.025 for 0.5, 0.75, and 1 mM, respectively; Table 3). Conversely, picrotoxin treatment was not predicted to have any impact on the capture proportion of Wol + flies (*P* = 0.433, 0.939, 0.504 for 0.5, 0.75, and 1 mM, respectively; Table 3). Additionally, the GLM did not predict any significant effects of infection or picrotoxin treatment for capture proportion in unbaited traps (*P* > 0.05 for all comparisons; Table 3). Taken together, these data indicate that the *Wolbachia*-dependent increase in olfactory-cued capture seen in mated females is unaffected by the inhibition of GABA_A_-mediated transmission by picrotoxin.

## DISCUSSION

In this study, we investigated the interactions of *Wolbachia* and the somatic tissues of *D. melanogaster* with a focus on the brain. We identified multiple protein changes within the heads and bodies of infected flies, and we found that glutamic acid decarboxylase was specifically modified within the head upon infection. Motivated by the “Behavioral Manipulation Hypothesis” (34), we proposed that *Wolbachia* in the brain of *D. melanogaster* may cause this change in GAD to manipulate neural function and elicit beneficial changes in host behavior. In support of this hypothesis, we discovered an improved olfactory response to food odors in mated female *Drosophila* upon infection. Given the importance of this behavior for food scavenging and oviposition (35), an enhanced ability to find food sources could make *Wolbachia*-infected females more competitive within an environment than their uninfected counterparts. This scenario would consequently benefit *Wolbachia*, as a more fit host would have more offspring for the bacterium to be passed onto. To connect our proteomic and behavioral phenotypes, we examined whether GABA levels changed upon infection. However, we did not observe any changes in GABA production within infected flies. Additionally, our treatments with picrotoxin revealed that the *Wolbachia*-induced olfactory change in mated females is not dependent on signaling through GABA_A_ receptors. Therefore, we cannot conclude that *Wolbachia* is eliciting a change in GAD to cause a change in host behavior. However, the results of our work provide new insights into the *Wolbachia–Drosophila* relationship overall.

Our work provides the first description of post-translational modifications that occur during *Wolbachia* infection within a host. The characterization of PTMs during infection is an important step in understanding host–microbe interactions more comprehensively, as PTMs regulate the function, stability, localization, and interaction of proteins (36). These modifications have been shown to be important in the life cycle of many intracellular bacteria as well as the host response to such microbes (37, 38). For example, host immune responses typically entail the activation of kinase cascades, such as the MAPK and NF-κB signaling pathways. Bacteria have been shown to target the phosphorylation in these pathways, such as *Yersinia* species, which express the acetyltransferase YopJ (38, 39). This enzyme acetylates MAPKK6, thereby inhibiting phosphorylation (and activation) of the kinase, stopping the MAPK pathway in the host cell (39). Other bacteria, such as *Legionella*, use PTMs to create a more favorable niche for survival within the host (37, 40). *Legionella pneumophila* has been shown to express the effector protein LegK2, which phosphorylates the host protein complex ARP2/3. This PTM leads to remodeling of the actin cytoskeleton and prevents lysosomal interactions with the bacterial vacuole, thus creating a safe niche for the bacteria to replicate in (40). By studying the PTMs elicited by *Wolbachia* infection, we can gain key insights into the host*–Wolbachia* interface and the mechanisms through which this endosymbiont thrives.

**Fig 5:**
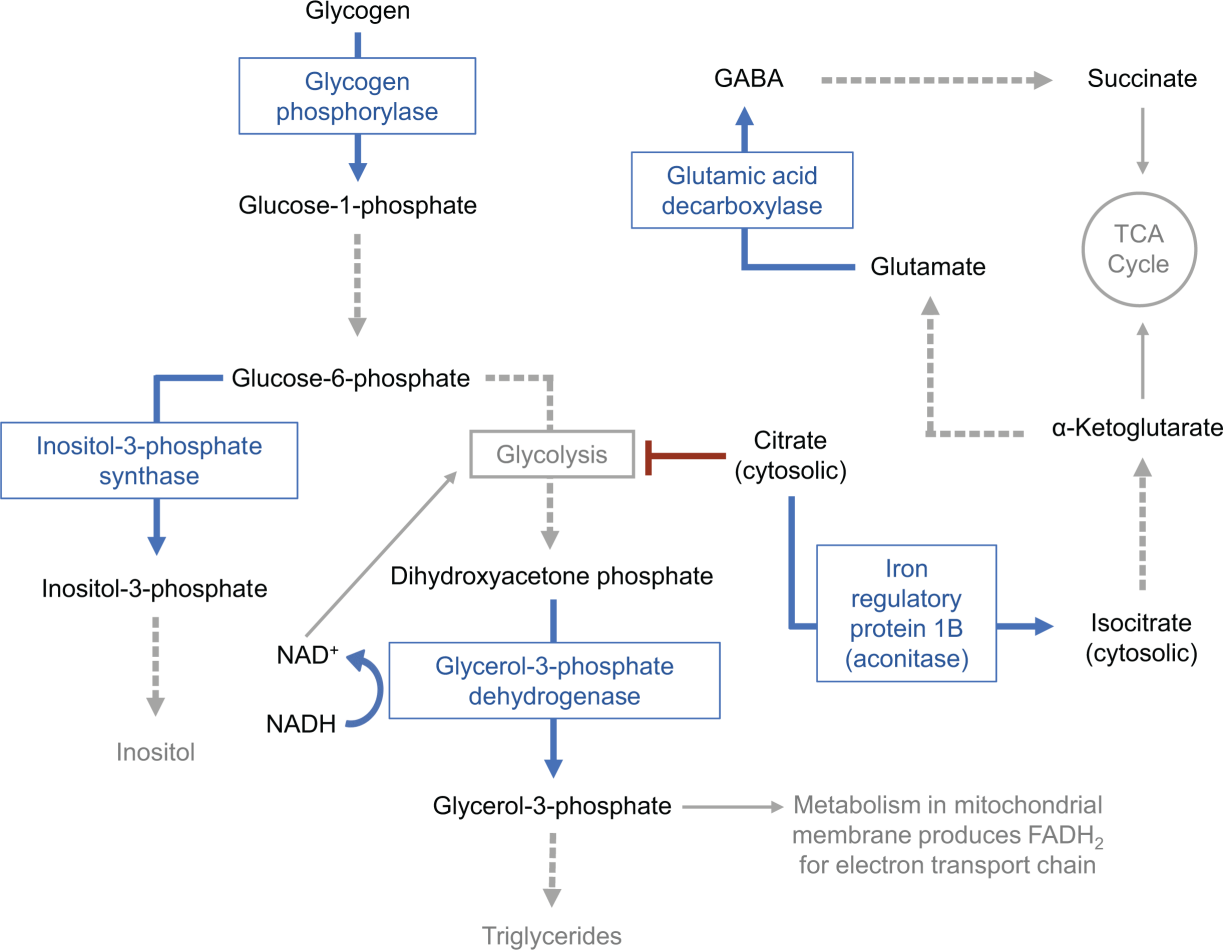
Metabolic pathways involving proteins differentially modified during *Wolbachia* infection in *D. melanogaster*. Proteins that undergo post-translational modification during infection and their known reactions are highlighted in blue. Dashed arrows represent pathways involving enzymes that were not shown to change in *Wolbachia-infected* flies in this study.

Of the six proteins differentially modified upon *Wolbachia* infection, five have direct connections to carbohydrate metabolism and energy production (Fig. 5). *Wolbachia*’s effect on these processes has been previously shown, as Baldridge et al. (2017) demonstrated elevated levels of proteins related to glycogen synthesis and turnover in an infected mosquito cell line (18). Enzymes related to glycolysis and the tricarboxylic acid (TCA) cycle were also upregulated in infected cells (18). *Wolbachia* is known to encode sugar transporters (41) and could be depleting host glucose levels within cells, thus creating the need for increased glycogen catabolism and subsequent carbohydrate processing. Accordingly, glycogen and glucose metabolism has also been shown to be crucial for *Wolbachia* fitness within filarial nematodes, indicating that the bacterium relies on host metabolism of sugars (42). Our work expands on these findings by demonstrating these changes in the *Drosophila* host as well as identifying post-translational modifications of the proteins instead of abundance changes.

In addition to understanding the *Wolbachia*–host relationship, our findings also pose new questions about *Drosophila* biology. The shift we observe in the GAD isoelectric point upon infection is reminiscent of a phosphorylation event, but the specific PTM occurring during infection is yet to be identified. Post-translational modification has not been described for *Drosophila* GAD, but both forms of human GAD are known to be phosphorylated (43). Human GAD67 is phosphorylated by PKA at threonine 91 to inactivate the enzyme, while GAD65 is phosphorylated by PKC at threonine 95 to activate GABA synthesis (43). *Drosophila* and human GADs share significant sequence homology, but the residues known to be modified in human GAD are not conserved in *Drosophila* GAD (44). Thus, GAD is being modified in some way during infection, but we cannot infer how this will affect enzymatic activity during *Wolbachia* infection. We did not detect a change in GABA levels when GAD was modified upon *Wolbachia* infection *in vivo*, but a more refined approach might be needed to investigate enzyme function further. For example, the modification of GAD could be elicited *in vitro*, and the subsequent effects on enzyme activity could be assessed using known assays (45). Additionally, it might be the case that modification of dGAD does not change its activity but rather its localization, similar to how GAD65 in humans is trafficked to synaptic vesicles upon phosphorylation (43). Differential localization of dGAD within neurons could lead to differential levels of intracellular GABA, which could be important for GABA metabolism. By uncovering the mechanism and function of GAD modification in *Drosophila*, we can discover more basic information about GABA production and processing in the *Drosophila* brain.

Differences in GAD activity upon *Wolbachia* infection could have considerable effects on the host and/or microbe. In addition to the regulation of behaviors in *Drosophila*, GABA is known to have roles in metabolism. On a systems level, GABA has been shown to regulate insulin signaling within the brain (46, 47). There is a known set of insulin-producing cells (IPCs) in the *Drosophila* brain that express the GABA_B_ receptor, and it has been shown that GABAergic neurons converge around these cells (46, 47). Knocking down the GABA receptor in these cells led to increased sensitivity to metabolic stresses and an increase in insulin-like peptide production (46). Thus, GABA signaling to IPCs is important for the regulation of insulin signaling. Interestingly, insulin signaling has also been implicated in the olfactory response to food odor (48). When insulin signaling is low, olfactory sensory neurons are activated to send information up the olfactory information pathway to initiate foraging for food. When insulin levels increase (such as after a meal), olfactory sensory neurons are inactivated, so foraging signals decrease (48). Therefore, GABA signaling to IPCs could also affect a fly’s olfactory response to food odors. This logic could provide additional explanation to how *Wolbachia* could be changing olfactory response in females. *Wolbachia* has been shown to increase insulin signaling in *Drosophila* to possibly modulate host metabolism to favor infection (21, 49), so it is possible that the bacterium uses the manipulation of GABA signaling to these IPCs to regulate insulin signaling in its host, which would lead to a change in food-finding olfactory behaviors. Additionally, because IPCs do not express GABA_A_ receptors (46), they would not have been affected by picrotoxin treatment during our experiments. Further investigation of *Wolbachia* and GABA in the cells surrounding IPCs is needed to confirm this hypothesis.

GABA is also known to have metabolic functions on the cellular level. In plants and microorganisms, GABA production and processing have been shown as a response to stress (25). In the GABA shunt pathway, intracellular GABA is catabolized by the enzymes GABA transaminase (GABA-T) and succinic semialdehyde dehydrogenase (SSADH) to produce succinate (25, 50). Succinate can then be shuttled into the TCA cycle to produce cellular energy and other metabolic intermediates (Fig. 5). This process has been identified in various bacterial species, including *E. coli* and *Listeria* (25). The intracellular parasite *Toxoplasma* was also shown to utilize this pathway in order to disseminate into neural tissues (51). A search through the *Wolbachia* genome (GenBank: AE017196.1) reveals no enzymes of the GABA shunt are encoded by the bacterium. However, *Wolbachia* could be affecting the metabolic processes of the host cell to create more favorable conditions for infection. The GABA shunt and TCA cycle primarily take place in mitochondria, and *Wolbachia* was recently shown to impact the mitochondrial metabolism of *Drosophila* cells (21). One 2020 study investigating changes in GABA metabolism by mitochondria showed that even though GABA levels were changing within the cell and within the mitochondria, total GABA levels within the *Drosophila* brain were not affected (52). Therefore, if *Wolbachia* is changing the mitochondrial metabolism of GABA, we would not be able to detect it with the quantification methods performed here. Thus, a more thorough investigation into GABA levels within different cellular compartments during *Wolbachia* infection is needed.

*Wolbachia’s* interaction with somatic tissues is largely understudied, and there is much to be learned about the host–microbe relationship within these tissues, particularly the nervous system. While our results do not confirm the “Behavioral Manipulation Hypothesis” behind *Wolbachia’s* localization in the nervous system, we have uncovered how this bacterium may adapt to the specialized niche of the *Drosophila* brain by modifying host metabolism. It could also be hypothesized that the behavioral effects seen during *Wolbachia* infection may be a consequence of this metabolic alteration. Continued investigation is needed to validate these hypotheses and to further understand how this master manipulator interacts with its host.

## MATERIALS AND METHODS

### Fly stocks and husbandry

The w^1118^ stocks (both uninfected and infected with the *Wolbachia* strain *w*Mel) were obtained from Dr. William Sullivan. Flies were reared on standard cornmeal agar medium. All stocks were kept at 25°C on a 12 h light–dark cycle. All flies were collected on the day of eclosion, then allowed to mature for 4–5 days before use. Females were placed into vials either with other females or males to obtain age-matched virgin and mated females.

### Two-dimensional difference gel electrophoresis (2D-DIGE)

Heads were removed from flies (separated by sex, mating status, and infection status) and rinsed with cold 1× PBS. Lysates were prepared by adding 100 µL of lysis buffer (7 M Urea, 2 M Thiourea, 10 mM HEPES pH 8.0, 10 mM DTT, 4% CHAPS) to either 70 heads or 30 bodies and manually homogenizing with a polypropylene pellet pestle (Kimble). Apart from sex-specific experiments, equal numbers of male and female flies were added to lysates. Homogenates were centrifuged at 4°C for 10 min at 15,000 rpm, and the supernatants were collected and stored at −80°C. Protein concentration in each lysate was determined via Bradford assay. One hundred micrograms of protein from each sample was labeled with Cy3- and Cy5-NHS minimal-labeling DIGE dyes (GE Healthcare). For every sample pair, a technical replicate was prepared in which the labeling scheme was reversed to account for any dye-dependent effects on protein migration. Two-dimensional gel electrophoresis was performed as previously described (26). After electrophoresis, gels were fixed in a solution of 40% methanol and 10% acetic acid before imaging on a custom-built gel imager. Due to the large size of these gels (18 × 18 cm), the gels were imaged in sections (4 × 4 cm each), and these sections were concurrently tiled together into one gel image using custom-written software for the imager (information regarding the imager and associated software is available upon request). Resultant gel images were visualized using ImageJ, and the relative intensity of protein spots within each channel was measured via Source Extractor (SExtractor) software (27). A minimum of three biological replicates were run, and the protein spots that consistently displayed differences on the gels were selected for identification.

### Protein identification via liquid chromatography-mass spectrometry (LC-MS)

Proteins selected for identification were excised from 2D gels using an in-house robotic spot cutter. Gel pieces corresponding to the same protein from multiple gels were pooled into single tubes containing 1% acetic acid in ultra-pure HPLC water. The liquid was then drained from the tubes, and the gel pieces were snap frozen.

In-gel digestion of the proteins was performed by Dr. Svitlana Yablonska (Impact Proteomics). Gel pieces were thawed and washed with 0.1 M ammonium bicarbonate, then underwent reduction with dithiothreitol (DTT) and alkylation with iodoacetamide. Following a thorough wash with ultra-pure water, gel pieces were crushed with a polypropylene pestle and dehydrated using concentrated acetonitrile. The crushed pieces were then saturated in an ammonium bicarbonate solution containing ProMTag-labeled trypsin (Impact Proteomics). Trypsinization was carried out at 37°C for 2 h with 850 rpm shaking. Supernatant containing digested peptides was then incubated with ProMTag capture resin (Impact Proteomics) for 30 min at room temperature to remove trypsin. Following elution and acidification with 1M formic acid, the peptides were lyophilized using a rotary evaporator.

LC-MS analysis of peptides was performed by Dr. Xi Peng and Dr. Kunhong Xiao (Allegheny Health Network Cancer Institute). Each sample was desalted using an Evotip Pure C18 disposable tip (EV2011, Evosep) following the manufacturer’s protocol. With an Evosep One HPLC (Evosep), the desalted peptides were eluted off the Evotip and loaded onto an Evosep EV1109 performance analytical column (8 cm × 150 µm inner diameter, 1.5 µm ReproSil Saphir C18 beads). Peptide separation was carried out according to the manufacturer’s preset 11.5 minute, 100 samples-per-day (SPD) method with 0.1% formic acid in water as solvent A and 0.1% formic acid in acetonitrile as solvent B. All mass spectrometric data were collected with a timsTOF Pro 2 mass spectrometer operated in the positive mode with TIMS enabled. A data-dependent acquisition with parallel accumulation-serial fragmentation (DDA-PASEF) method was utilized. Briefly, a full scan was first acquired for the mass range of *m/z* 100 to 1,700, with the TIMS 1/k0 window set as 0.60–1.60 V·s/cm^2^. For a 100% duty cycle (1.17 s cycle time) at a ramp rate of 9.42 Hz, the ramp time and accumulation time were set to 100 ms respectively. The precursor isolation window was set to be linear across the *m/z* range, with a width of 2 *m/z* at 700 *m/z* and 3 *m/z* at 800 *m/z*. Precursors with charge states up to +5 that passed the intensity threshold (2.5E3) were then selected for fragmentation. Ten PASEF ramps were allowed during each cycle, with a dynamic exclusion duration of 0.4 min. Nitrogen was used as the collision gas, and the collision energy ranged from 20 eV to 59 eV across the defined TIMS 1/k0 window.

The MS data were searched with the Bruker Parallel Search Engine in Real-time (PaSER) platform against a reviewed *Drosophila* protein database from Uniprot. Enzyme activity was set to be fully tryptic, with up to two missed cleavages allowed. The following variable modifications were considered: oxidation on M (+15.994915 Da), phosphorylation on S/T/Y (+79.966331 Da), and carbamidomethyl on C (+57.021464 Da), with up to two modification sites allowed for each peptide. Protein false discovery rate of 1% was applied. The mass tolerance for both the precursors and fragments was set to ±20 ppm, with each protein requiring at least one peptide identified within a mass error of ±10 ppm. Additional post-search filters were applied to the peptides. XCorr score cutoff was set to 1.0 for peptides with a charge state of +1 and 0.8 for peptides with charge states of +2 to +4. DeltaCN cutoff was set to 0.1 for all peptides. A minimum percentage of identified b/y ions was set to 40%.

### Fluorescence-coupled assay for GABA quantification

Total GABA content of *D. melanogaster* heads was measured using an assay adapted from the work of Ippolito and Piwnica-Worms (53) in which the metabolism of GABA is used to drive the synthesis of the fluorescent compound resorufin. To prepare solutions of GABA fly heads, flies were separated by sex, mating status, and infection status, and their heads were removed and rinsed with cold 1× PBS. Lysates were prepared by adding 150 µL of lysis solution (50 mM NaOH, 1 mM EDTA) to 100 heads and manually homogenizing with a polypropylene pellet pestle (Kimble). Homogenates were centrifuged at 4°C for 10 min at 15,000 rpm, and the supernatants were collected. A small volume of lysate (15 µL) was taken at this step for protein quantification via Bradford assay. To the remaining lysates, an equal volume of 100 mM HCl was added, and the samples incubated at 60°C for 30 min. The pH was then brought up to 8.0 using 400 mM Tris solution. Following another centrifugation (15,000 rpm for 5 min at 4°C), supernatants were collected and either used in the assay immediately or frozen in liquid nitrogen and stored at −80°C. Standard solutions of GABA (Sigma, #A2129) ranging from 0.5 µM to 500 µM were prepared in lysis solution, then processed and stored in an identical manner to the head lysates.

All components of the reaction mixture were obtained from Sigma: GABase (#G7509), diaphorase (#D5540), resazurin (#R7017), nicotinamide adenine dinucleotide phosphate (NADP; #481972), α-ketoglutarate (α-KG; #75890), dithiothreitol (DTT; #D0632), and 2-aminoethyl hydrogen sulfate (AEHS; #06720). The reaction mixture was prepared as a master mix and was made fresh each time the assay was conducted. The solution contained 0.063 U/ml GABase, 0.063 U/ml diaphorase, 6.25 µM resazurin, 100 µM NADP, 5 mM alpha-ketoglutarate, and 3.125 µM DTT in 100 mM Tris base (pH 8.8). An identical solution was prepared with the addition of AEHS (50 mM final concentration), and both reaction mixtures were kept at room temperature for at least 15 min before assay start. Reaction mixtures were loaded into a 96-well plate (10 µL standards and samples + 90 µL reaction master mix in each well), and the fluorescence of resorufin was measured via a Tecan Spark plate reader every 30 s for 45 min (544 nm excitation, 590 nm emission). Each sample and standard were assayed with and without AEHS in the reaction mixture.

To calculate the amount of GABA in each sample, the fluorescence values from reactions containing AEHS were subtracted from the fluorescence values from reactions without AEHS at each time point. The normalized fluorescence over time was plotted for each GABA standard, and the initial slope of the resulting curves (fluorescence over time for the first 600 s of the assay) was then determined. Initial slope was plotted against the concentration of GABA standards to create a standard curve. The initial slope of normalized fluorescence value over time was then calculated for each unknown sample, and the equation of the standard curve was used to calculate the concentration of GABA in each sample. The concentration of GABA was then normalized against the concentration of protein in each sample. The assay was run with three biological replicates and two technical replicates of each sample.

### Immunohistochemistry

Brains were dissected and immunostained as previously described (54). Briefly, brains were dissected in PBST (0.3% Triton-X in phosphate-buffered saline) then fixed in a solution of PBST containing 4% formaldehyde for 20 min at room temperature. After three washes (20 min each) with PBST, the brains were blocked in a solution of 5% normal goat serum (Abcam) in PBST for 1.5 h at room temperature. Brains were then transferred to solutions of primary antibodies in PBST containing 5% NGS and incubated at 4°C for 2 days. All samples were stained with a polyclonal rabbit antibody against GABA (Sigma #A2052; 1:1000) and a monoclonal mouse antibody against ELAV (DSHB Hybridoma Product Elav-9F8A9; 1:25). Brains were then washed three times with PBST and moved to a solution of secondary antibodies in PBST containing 5% NGS. All samples were incubated with goat anti-rabbit antibody conjugated to Alexa Fluor 568 (Abcam, ab175471; 1:1,000) and goat anti-mouse antibody conjugated to Cy5 (Abcam, ab6563; 1:1,000). Brains incubated in secondary antibody solution at 4°C for 1 day then washed three times with PBST and once with PBS. For comparison of staining in *Wolbachia*-positive and -negative samples, antibody solutions were prepared in one tube to ensure all samples received the same concentrations of reagents. Samples were mounted in Anti-Fade Fluorescence Mounting Medium (Abcam, ab104135) on a positively charged slide flanked by two #1 coverslips. Brains were mounted such that antennal lobes were facing up. All microscopy was performed on a Zeiss Axio Observer Z1 confocal microscope using a 20× or 63× objective. Image acquisition settings (laser power, exposure time, etc.) were kept consistent for all samples.

### Image analysis

All images were processed via ImageJ. For fluorescence quantification, the selection tool was used to outline the brain throughout the entire confocal stack using the ELAV staining as a guide, and the mean intensity of the selected area was measured in each channel. Measurements were exported to Microsoft Excel for further calculations. The mean intensity of the GABA channel was always normalized against the mean intensity of the ELAV channel.

### Olfactory trap assay

Odor-baited traps were adapted from those described by Woodard, et al (1989) (55). Each trap was constructed from one 1.5 mL microfuge tube and one pipette tip (Fisherbrand SureOne beveled pipette tips, 1–200 μL). The microfuge tube was cut approximately 4 mm from its bottom, creating an opening 5 mm in diameter. The pipette tip was cut along the first gradation (1 cm from the tip) to create an opening 2 mm in diameter. The smaller end of the pipette tip was inserted snugly into the smaller end of the microfuge tube. The cap of the microfuge tube was filled with yeast paste (0.5 g/mL Red Star active dry yeast in distilled water). Unbaited control traps were constructed in an identical manner but were not filled with yeast paste. Traps were placed in petri dishes (100 × 15 mm) containing approximately 20 mL of 1.5% agarose for the assays.

Flies were separated by infection status and sex before the assay to mitigate the effects of pheromones on olfactory performance. These groups were starved in empty vials for 2 h. Then, following a brief anesthetization on ice, 25 flies were introduced into each agarose plate with a trap. Each plate was sealed with Parafilm to retain humidity and to keep flies from escaping during the assay period. Plates were placed under red light in a temperature-controlled area, and fly activity was recorded for 12 h using a Retiga LUMO 16-bit CCD camera (1 frame per 30 min). Experiments were consistently conducted in the afternoon, with recording beginning between ZT5 and ZT6 in the flies’ light/dark cycle.

All assay recordings were scored blind. For every frame, the number of flies that had entered the trap was counted. Capture proportion was calculated by dividing the number of captured flies by the total number of flies in the arena. Flies that died during or had limited mobility throughout the assay were excluded from these data.

### Picrotoxin treatment

Picrotoxin was obtained from Sigma (product no. P1675). A 100 mg/mL stock solution in DMF was prepared and stored at −20°C. On the day of treatment, stocks were diluted to their final concentration with solutions containing 5% sucrose and 10% red food color (McCormick). Control solutions containing 5% sucrose, 10% dye, and identical amounts of DMF were also prepared. Wicks of filter paper (approximately 1 cm^2^) were saturated with the treatment and control solutions and were placed in empty vials. Flies were starved for 2 h, as in the untreated assays, then moved to the treatment vials for 90 min. Flies were then briefly anesthetized on ice, and the flies that consumed the treatment solution (characterized by red abdomens) were selected and introduced into the agarose plates with olfactory traps.

### Statistical analyses

All data analyses were performed using Microsoft Excel and R (v 4.3.0). Moreover, *t*-tests were used to compare log_2_ ratios of Wol− and Wol + proteins for spots on DIGE gels. To determine differences in GABA levels within *Drosophila* brains, *t*-tests were performed on both enzymatic and imaging data (pairwise between groups with Bonferroni adjustment for multiple comparisons). To analyze capture data from the initial odor-baited trap assays, we utilized a Cox proportional hazards model using the “survminer” package in R. Capture data were stratified by sex in order to satisfy the proportional hazard assumption (Surv(Time, Status) ~Infection + strata(Sex)). A Cox proportional hazards model was also attempted for the capture data from picrotoxin-treated flies, but the “treatment” factor violated the proportional hazards assumption (*P* < 0.05 for Schoenfeld residuals test). Therefore, to test the effect of treatment and infection on capture data, we performed a generalized linear model (GLM) with binomial distribution and logit function in R. The effects of infection and treatment on capture were tested both separately and as interacting factors. For all odor-baited trap assay data, capture information from yeast-baited and unbaited traps was analyzed separately.

## Data Availability

The mass spectrometry proteomics data produced in this study have been deposited to the ProteomeXchange Consortium via the PRIDE (56) partner repository with the dataset identifier PXD055815.

## SUPPLEMENTAL MATERIAL

Fig. S1 spectrum.02465-24-s0001.eps Capture proportion of all flies (males, mated females, and virgin females) in odor-baited trap assays.

Supplemental material spectrum.02465-24-s0002.docx Tables S1 and S2.

ASM does not own the copyrights to Supplemental Material that may be linked to, or accessed through, an article. The authors have granted ASM a non-exclusive, world-wide license to publish the Supplemental Material files. Please contact the corresponding author directly for reuse.
