# Supplementary material for: Wolbachia infection confers post-translational modification of glutamic acid decarboxylase and other proteins in D. melanogaster
Source: Microbiol Spectr. 2025 Apr 28;13(6):e02465-24. doi: 10.1128/spectrum.02465-24 (PMC12131856; doi:10.1128/spectrum.02465-24)
Supplement: Supplemental material — Tables S1 and S2. [file spectrum.02465-24-s0002.docx]

**Table S1.** Sex-specific quantification of *D. melanogaster* protein from *Wolbachia*-infected and uninfected heads in spots identified by 2D-DIGE analysis

| Protein ID | Gel Spot*^a^* | | Log_2_(Wol+/Wol-)*^b^* | | | | | |
| --- | --- | --- | --- | --- | --- | --- | --- | --- |
|  |  |  | Males | | Mated Females | | Virgin Females | |
|  |  |  | Average±SE | P-value*^c^* | Average±SE | P-value*^c^* | Average±SE | P-value*^c^* |
| Glycogen phosphorylase | A | 1 | 1.60 ±0.82 | 1.06x10^-6^ | 1.66 ±0.56 | 2.47x10^-5^ | 1.87 ±1.19 | 8.23x10^-5^ |
|  |  | 2 | 1.68 ±0.08 |  | 1.59 ±0.48 |  | 1.78 ±0.88 |  |
|  |  | 3 | -1.86 ±0.57 |  | -1.10 ±0.39 |  | -2.23 ±0.28 |  |
|  |  | 4 | -1.92 ±0.32 |  | -3.28 ±0.90 |  | -3.20 ±0.41 |  |
| Iron regulatory protein 1B | B | 1 | -1.19 ±0.33 | 3.94x10^-3^ | -1.20 ±0.57 | 0.084 | -0.83 ±0.08 | 0.019 |
|  |  | 2 | 0.54 ±0.29 |  | 0.10 ±0.60 |  | -0.03 ±0.14 |  |
| Inositol-3-phosphate synthase | C | 1 | 0.85 ±0.38 | 0.042 | 0.42 ±0.18 | 1.22x10^-3^ | -0.47 ±0.40 | 0.251 |
|  |  | 2 | -0.62 ±0.26 |  | -1.29 ±0.18 |  | 0.23 ±0.86 |  |
| Glutamic acid decarboxylase | D | 1 | 0.90 ±0.22 | 1.61x10^-3^ | 1.09 ±0.25 | 7.29x10^-5^ | 0.83 ±0.24 | 2.47x10^-3^ |
|  |  | 2 | -0.26 ±0.10 |  | -0.43 ±0.07 |  | -0.45 ±0.17 |  |
| Elongation factor 1-gamma | E | 1 | -2.14 ±0.01 | 4.11x10^-5^ | -0.75 ±0.16 | 2.80x10^-4^ | -0.76 ±0.24 | 2.23x10^-3^ |
|  |  | 2 | 1.05 ±0.13 |  | 0.87 ±0.28 |  | 1.95 ±0.22 |  |
| Glycerol-3-phosphate dehydrogenase | F | 1 | -0.80 ±0.32 | 3.5x10^-5^ | -1.07 ±0.09 | 3.53x10^-9^ | -1.58 ±0.60 | 1.32x10^-4^ |
|  |  | 2 | -0.46 ±0.61 |  | -0.72 ±0.34 |  | -1.47 ±0.37 |  |
|  |  | 3 | 2.62 ±0.26 |  | 1.92 ±0.35 |  | 2.86 ±0.29 |  |
|  |  | 4 | 3.13 ±1.65 |  | 1.69 ±0.25 |  | 1.15 ±0.90 |  |

^a,b,c^ See notes on Table 2 for column descriptors.

**Table S2.** Results of the Cox proportional hazard model assessing the capture of *Wolbachia*-infected and uninfected *D. melanogaster* in odor-baited trap assays

| Time | Trap Type | Subset | Coefficient | Hazard Ratio | SE(Coef) | Z-value | P-value |
| --- | --- | --- | --- | --- | --- | --- | --- |
| 12 hours | Yeast-Baited Traps | All | 0.7191 | 2.0526 | 0.1097 | 6.557 | 5.48E-11 |
|  |  | Mated Females | 0.8985 | 2.4559 | 0.1449 | 6.199 | 5.69E-10 |
|  |  | Virgin Females | -0.01562 | 0.9845 | 0.24155 | -0.065 | 0.948 |
|  |  | Males | 0.9273 | 2.5276 | 0.2415 | 3.84 | 1.23E-04 |
|  | Unbaited Traps | All | 1.4994 | 4.4792 | 0.2353 | 6.373 | 1.85E-10 |
|  |  | Mated Females | 1.3606 | 3.8984 | 0.4277 | 3.181 | 0.00147 |
|  |  | Virgin Females | 0.9776 | 2.6581 | 0.3538 | 2.763 | 0.00573 |
|  |  | Males | 2.3857 | 10.8663 | 0.5272 | 4.525 | 6.04E-06 |
| 6 hours | Yeast-Baited Traps | All | 0.8887 | 2.432 | 0.1646 | 5.398 | 6.74E-08 |
|  |  | Mated Females | 0.8132 | 2.2552 | 0.1835 | 4.433 | 9.31E-06 |
|  |  | Virgin Females | 0.9108 | 2.4864 | 0.488 | 1.866 | 0.062 |
|  |  | Males | 1.5893 | 4.9003 | 0.6363 | 2.498 | 0.0125 |
|  | Unbaited Traps | All | 3.827 | 45.929 | 1.013 | 3.78 | 1.57E-04 |
|  |  | Mated Females | 2024 | 6.16E+08 | 8.13E+03 | 0.002 | 0.998 |
|  |  | Virgin Females | 3.107 | 22.345 | 1.028 | 3.023 | 0.0025 |
|  |  | Males | 2029 | 6.50E+08 | 6.77E+03 | 0.003 | 0.998 |
